# Burning Mouth Syndrome Among Postmenopausal Women: A Clinic-Based, Cross-Sectional Study

**DOI:** 10.7759/cureus.116879

**Published:** 2026-09-24

**Authors:** Ashwinirani S.R., Girish Suragimath, Disha G Suragimath

**Affiliations:** 1 Department of Oral Medicine and Radiology, School of Dental Sciences, Krishna Vishwa Vidyapeeth (Deemed to be University), Karad, IND; 2 Department of Periodontology, School of Dental Sciences, Krishna Vishwa Vidyapeeth (Deemed to be University), Karad, IND; 3 Department of Biochemistry, Krishna Vishwa Vidyapeeth (Deemed to be University), Karad, IND

**Keywords:** burning mouth syndrome, dry-mouth, post-menopausal symptoms, post-menopause management, prevalence, women health

## Abstract

Introduction

Menopause is a physiological transition associated with several systemic and oral changes. Burning mouth syndrome (BMS), characterized by persistent or recurrent intraoral burning in the absence of an identifiable local or systemic cause, predominantly affects middle-aged and postmenopausal women. This study aimed to determine the clinic-based frequency of BMS and describe its demographic and clinical characteristics among postmenopausal women attending an oral medicine clinic.

Materials and methods

This cross-sectional observational study was conducted at the Department of Oral Medicine and Radiology of the School of Dental Sciences, Krishna Vishwa Vidyapeeth, Maharashtra, India. A total of 1,230 postmenopausal women were screened between January 2025 and December 2025, of whom 278 were excluded because they did not meet the eligibility criteria or declined participation. The remaining 952 eligible participants were included in the analysis. Data regarding demographic variables, systemic comorbidities, medication history, and clinical characteristics of BMS, as well as associated subjective symptoms like oral dryness and taste disturbances, were recorded. Descriptive statistics and Pearson's chi-square test were used for analysis, with a* p* value <0.05 considered statistically significant.

Results

Of the study participants, 26.5% (n=252) had BMS, with the largest proportion of women belonging to the age group of 55-59 years. Hypertension was the most prevalent systemic comorbidity, followed by diabetes mellitus. BMS symptoms were most commonly reported during the daytime, with the tongue being the most commonly affected site. Among women with BMS, oral dryness was reported most frequently in the age group of 60-65 years, whereas taste disturbances were most frequently reported in the age group of 55-59 years.

Conclusion

BMS was observed in one-fourth of the postmenopausal participants, with the tongue being the most commonly affected site and oral dryness and taste disturbances being frequent accompanying symptoms. Given the single-centre, cross-sectional design, the findings should be interpreted within the context of the study population and cannot establish causal relationships. Early recognition, exclusion of secondary causes, and appropriate multidisciplinary management may facilitate better clinical care for affected postmenopausal women.

## Introduction

Menopause is a physiological process typically occurring in the fifth decade of female life. Menopause in women marks the end of reproductive years. It is defined as the absence of menstrual periods for 12 consecutive months and occurs when the ovaries significantly reduce the production of estrogen and progesterone. The various symptoms associated with menopause are hot flashes, changes in sleep patterns, night sweats, mood swings, and vaginal dryness [1]. Oral symptoms reported during menopause may include xerostomia, oral burning or altered oral sensation, and changes in periodontal tissues. Persistent or recurrent oral burning in the absence of an identifiable local or systemic cause meets the diagnostic criteria for burning mouth syndrome (BMS) [2]. Postmenopausal women may also suffer from osteoporosis, cardiovascular disease, breast cancer, cervix cancer [3].

The International Association for the Study of Pain has described BMS as a burning pain in the tongue and/or other oral mucous membranes in the absence of clinical signs or laboratory findings [4]. BMS is also known by several other terms, including oral dysesthesia, sore tongue, stomatodynia, and stomatopyrosis. The most affected area by BMS is the tongue, so it is named as glossodynia, glossalgia (painful tongue), and glossopyrosis (burning tongue). BMS is significantly more common among women than in men, with a reported ratio of 7:1, and it most frequently affects middle-aged, postmenopausal women [5]. The prevalence of BMS increases with age in both men and women, and predominantly affects women in the fifth to seventh decades of life [6]. The age-adjusted prevalence of BMS in women is significantly higher than in men: 168.6 vs 35.9 per 100,000 persons [7]. Several clinical conditions such as lichen planus, candidiasis, viral infections, and xerostomia also present with a burning sensation in the mouth, but these are typically accompanied by visible red or white lesions on the oral mucosa [8].

Evidence describing the clinic-based frequency and clinical characteristics of BMS among postmenopausal women in India remains limited. Therefore, the present study was undertaken to determine the frequency of BMS and describe its demographic and clinical characteristics among postmenopausal women attending an oral medicine clinic. The objective of this study was to evaluate the demographic and clinical characteristics of BMS, including site, age distribution, associated systemic conditions, and duration and pattern of burning symptoms among postmenopausal women.

## Materials and methods

Study design

This was a cross-sectional observational study conducted in the Department of Oral Medicine and Radiology, School of Dental Sciences, Krishna Vishwa Vidyapeeth, Karad, Maharashtra, India, from January 2025 to December 2025. The study was approved by the Institutional Ethics Committee of Krishna Vishwa Vidyapeeth (Deemed to be University) (reference number: KVV/IEC/04/2025) and was conducted in accordance with the ethical principles of the Declaration of Helsinki, as revised in October 2024. Written informed consent was obtained from all participants before enrollment. A flow chart of the study design is presented in Figure 1.

**Figure 1:**
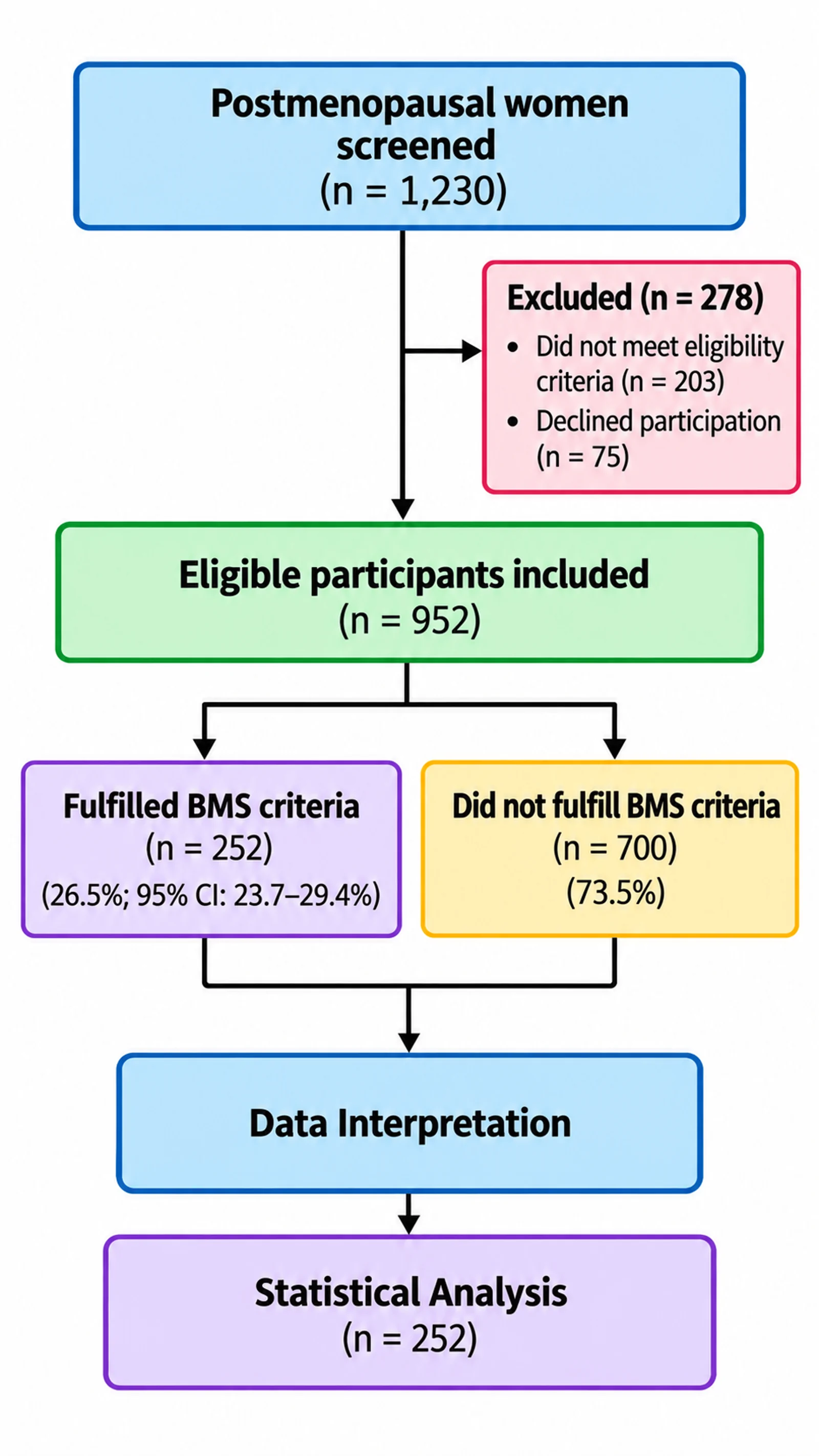
Flow diagram of the study design. BMS: burning mouth syndrome

Eligibility criteria

Women aged 48 years and above who had attained natural menopause and were willing to participate were enrolled in the study. Women were excluded if they had perimenopause or had not completed 12 consecutive months of amenorrhea, had surgically or medically induced menopause, were on current hormone replacement therapy (HRT), had neurological disorders capable of producing oral orofacial burning or neuropathic pain such as trigeminal neuralgia, or had an identifiable systemic or metabolic condition considered to provide a sufficient explanation for the burning symptoms. Women unable to reliably report symptoms or complete the study questionnaire because of cognitive or psychiatric impairment, those unable to understand the study procedures, and those who declined informed consent were also excluded.

Sample size and sampling method

A total of 1,230 postmenopausal women were screened during the one-year study period. Of these, 278 were excluded because they did not meet the eligibility criteria or declined participation. The remaining 952 eligible women who provided informed consent were included consecutively. Because the study used a fixed one-year recruitment period, no separate a priori sample-size calculation was performed. The final sample size comprised all eligible participants recruited consecutively during this period, and this approach was adopted to maximize the number of eligible participants available within the study period.

Diagnostic criteria for BMS

Demographic characteristics, medical history, systemic comorbidities, and medication use were recorded using a structured clinical proforma. All participants reporting oral burning symptoms underwent a comprehensive intraoral examination by a single experienced oral medicine specialist using a mouth mirror and probe. The oral mucosa, tongue, palate, buccal mucosa, gingiva, and other intraoral structures were examined to identify clinically evident lesions or local conditions that could account for the symptoms. A detailed medical and medication history was obtained to identify potential systemic or drug-related causes of oral burning.

The diagnosis of BMS was established according to the International Classification of Orofacial Pain (ICOP) criteria, which define BMS as a daily or recurrent intraoral burning sensation occurring for more than two hours per day for more than three months, in the absence of a clinically evident causative lesion or identifiable local or systemic cause [9]. The diagnosis incorporated clinical examination, medical and medication history, and exclusion of clinically identifiable local and systemic causes. Where clinically indicated, investigations such as glucose/HbA1c, hematological parameters, thyroid function, evaluation for candidal infection, and salivary function were advised. Participants in whom an identifiable condition was considered sufficient to explain the burning symptoms were not classified as having primary BMS. Conditions such as diabetes mellitus, hypertension, cardiovascular disease, and renal disease, along with relevant medications, were recorded as clinical variables and were not considered exclusion criteria per se.

BMS characteristics

BMS patients were classified based on the site, duration, frequency, and timing of the burning sensation. The duration of symptoms was categorized as: 3-6 months, >6 months to 1 year, >1 year to 2 years, and above 2 years. The frequency of burning was recorded as intermittent and seldom, intermittent and often, or continuous. The timing of the symptom occurrence was documented as morning, evening, daytime, nighttime, or both day and night. Additionally, subjective symptoms such as oral dryness and taste disturbances were also recorded.

Stress level estimation

Stress levels among postmenopausal women suffering from BMS were assessed using the Perceived Stress Scale (PSS-10) [10], a widely used psychological tool for measuring perceived stress. The PSS-10 consists of 10 items that assess both the positive and negative aspects of perceived stress. Each item is rated on a five-point Likert scale ranging from 0 to 4, where 0 indicates “never”, 1 “rarely”, 2 “sometimes”, 3 “often”, and 4 “always”. Items 4, 5, 7, and 8 were reverse-scored (0=4, 1=3, 2=2, 3=1, 4=0), and item scores were summed to obtain the total PSS-10 score. Based on the total score, stress levels were categorized as follows: Low (0-13), Moderate (14-26), and High (27-40). In this study, the Marathi version of the PSS-10 questionnaire was used to facilitate ease of understanding among the participants and was administered using a structured, face-to-face, one-to-one interview to facilitate accurate and consistent responses [11].

Statistical analysis

Data were entered, coded, and tabulated using Microsoft Excel (Microsoft Corporation, Redmond, Washington, United States) and subsequently analyzed using the IBM SPSS Statistics for Windows, version 21.0 (IBM Corp., Armonk, New York, United States). Descriptive statistics were used to summarize the demographic characteristics, systemic comorbidities, medication history, and clinical characteristics of the study participants. Categorical variables were expressed as frequencies and percentages. Associations between categorical variables were evaluated using the Pearson chi-square test, as appropriate. The frequency of BMS was calculated as the proportion of participants who fulfilled the predefined diagnostic criteria for BMS among the total number of eligible postmenopausal women assessed. All statistical tests were two-tailed, and a *p* value <0.05 was considered statistically significant.

## Results

A total of 952 postmenopausal women were selected and examined during the study period. Of these, BMS was identified in 252 participants (26.5%; 95% CI: 23.7-29.4%). The remaining 700 participants did not fulfill the predefined diagnostic criteria for BMS. The demographic and clinical characteristics of the participants with BMS are presented in Table 1. The largest proportion of BMS patients belonged to the age group of 55-59 years (29.4%), followed by the age group of 60-65 years (25.4%). Hypertension was the most frequently reported systemic condition (30.2%), followed by diabetes mellitus (24.6%). Antihypertensive medication was the most frequently reported medication category (24.6%). The burning sensation was most commonly reported during daytime and was predominantly intermittent and often in nature. The tongue was the most commonly affected site, followed by the palate. The largest proportion of women with BMS experienced it for 6-12 months (Table 1).

**Table 1:** Demographic data and clinical characteristics of BMS patients (N=252) Note: Participants with more than one systemic condition were classified under “Multiple systemic conditions”, and those taking medications from more than one therapeutic class were categorized under "Multiple medication use".  Therefore, the categories within systemic conditions and medication use are mutually exclusive. BMS: burning mouth syndrome

Parameters	Category	Frequency	Percentage
Age in years	<50	12	4.8%
	50-54	52	20.6%
	55-59	74	29.4%
	60-65	64	25.4%
	>65	50	19.8%
Systemic condition	Diabetes mellitus	62	24.6%
	Cardiovascular disease	18	7.1%
	Hypertension	76	30.2%
	Kidney disease	8	3.2%
	Multiple systemic conditions	11	4.4%
	None	77	30.6%
Medication use	Antihypertensive drugs	62	24.6%
	Cardiovascular medications	16	6.3%
	Kidney-related medications	8	3.2%
	Anticoagulants	21	8.3%
	Antidiabetic drugs	52	20.6%
	Multiple medications use	13	5.2%
	No medication use	80	31.7%
Time of occurrence of BMS symptoms	Morning	12	4.8%
	Night	32	12.7%
	Day and night	40	15.9%
	Daytime	99	39.3%
	Evening	69	27.4%
Frequency of BMS	Intermittent and seldom	61	24.2%
	Intermittent and often	156	61.9%
	Continuous	35	13.9%
Site of BMS	Tongue	108	42.9%
	Palate	80	31.7%
	Cheeks	29	11.5%
	Entire oral cavity	35	13.9%
Duration of BMS	3-6 months	34	13.5%
	>6 months to 1 year	103	40.9%
	>1 to 2 years	61	24.2%
	>2 years	54	21.4%

Table 2 presents the distribution of oral dryness and taste disturbances among BMS patients according to age group. Oral dryness was reported by 121 (48.0%) of the 252 BMS patients. The highest proportion of reported oral dryness was observed among women aged 60-65 years (62/64, 96.9%), followed by those aged <50 years (11/12, 91.7%). The lowest proportion was observed in the 55-59-year age group (15/74, 20.3%). A statistically significant association was observed between age group and oral dryness (χ²=103.43, df=4, *p*<0.0001). Taste disturbances were reported by 159 (63.1%) BMS patients and were most frequent among those aged 55-59 years (68/74, 91.9%), followed by those aged 60-65 years (45/64, 70.3%). The lowest proportion was observed among patients aged 50-54 years (13/52, 25.0%). Age group was also significantly associated with taste disturbances (χ²=63.64, df=4, *p*<0.0001).

**Table 2:** Association of age group with oral dryness and taste disturbances among patients suffering from BMS. Note: Values are expressed as frequency (n) and percentage (%). Percentages for symptom presence and absence were calculated using the total number of BMS patients within each respective age group as the denominator. Associations between age group and each subjective symptom were assessed using Pearson's chi-square test.  **Statistically significant.

Subjective symptoms	Age (years)	Total patients with BMS in age group	Symptom present, n (%)	Symptom absent, n (%)	*p*-value
Oral dryness	<50	12	11 (91.7%)	1 (8.3%)	<0.0001**
	50–54	52	18 (34.6%)	34 (65.4%)	
	55–59	74	15 (20.3%)	59 (79.7%)	
	60–65	64	62 (96.9%)	2 (3.1%)	
	>65	50	15 (30.0%)	35 (70.0%)	
Total		252	121 (48.0%)	131 (52.0%)	
Taste disturbances	<50	12	5 (41.7%)	7 (58.3%)	<0.0001**
	50–54	52	13 (25.0%)	39 (75.0%)	
	55–59	74	68 (91.9%)	6 (8.1%)	
	60–65	64	45 (70.3%)	19 (29.7%)	
	>65	50	28 (56.0%)	22 (44.0%)	
Total		252	159 (63.1%)	93 (36.9%)	

Table 3 presents the distribution of perceived stress levels among participants with BMS. Among the 252 participants with BMS, 135 (53.6%) had moderate perceived stress, while 69 (27.4%) had low perceived stress. High perceived stress was observed in 48 (19.0%) participants.

**Table 3:** Distribution of perceived stress levels among participants with BMS (N=252) BMS: burning mouth syndrome

Perceived stress	Frequency	Percentage
Low (0-13)	69	27.4%
Moderate (14-26)	135	53.6%
High (27-40)	48	19.0%

## Discussion

Lamey and Lewis categorized BMS into three categories based on fluctuations in pain severity over 24 hours [12]. Type 1 typically has no symptoms on waking and progressively worsens throughout the day, with variable nighttime symptoms. It may be related to nutritional deficiency or endocrine conditions such as diabetes mellitus. Type 2 is associated with chronic anxiety and displays symptoms throughout the day. Type 3 displays intermittent daytime symptoms and may have periods without any symptoms [13]. Food allergy is thought to be a potential underlying mechanism. The etiology of BMS is not fully understood and is considered to be multifactorial. Several theories suggest that estrogen may play a significant role in its development. Reduced estrogen levels can lead to atrophy of the oral mucosa, increasing susceptibility to inflammation and contributing to BMS symptoms [14]. In a rat model, Bereiter et al. demonstrated that estrogen may influence the sensory and autonomic components of craniofacial pain in a sex-dependent manner, possibly through its effects on trigeminal ganglion neurons and the caudal regions of the trigeminal subnucleus caudalis [15]. Image analysis and immunohistochemical studies have demonstrated a significant increase in P2X purinoceptor 3 receptor-expressing nerve fibers in patients with BMS, suggesting that these fibers may contribute to the sensory symptoms associated with the condition [16].

The present study found the frequency of BMS to be 26.5% in the study sample. This was comparable to estimates reported in studies involving postmenopausal or clinical populations, although differences in diagnostic criteria, sampling methods, and study settings limit direct comparison [17]. The study conducted by Malik et al. reported a prevalence rate of nearly 21% for BMS [18]. A study conducted by Gupta and Gupta [19] in Uttar Pradesh, India, showed a slightly higher prevalence rate compared to our study. A systematic review and meta-analysis by Wu et al. reported a pooled BMS prevalence of 1.73% in the general population and 7.72% among clinical patients, with a higher prevalence among women and individuals over 50 years of age [6]. The higher prevalence observed in the present study may partly be explained by the study population being restricted specifically to postmenopausal women, a group recognized to have a greater susceptibility to BMS, attending a single oral medicine clinic, whereas the meta-analysis included heterogeneous populations and study settings.

In the present study, the largest proportion of patients with BMS belonged to the age group of 55-59 years, followed by the age group of 60-65 years. This is consistent with the systematic review and meta-analysis by Wu et al., who found that BMS prevalence was higher among individuals older than 50 years [6]. A study conducted by Gurvits and Tan concluded that BMS can affect any age group between 27and 87 years; however, the average age of reference was 61 years [5]. The study conducted by Gupta and Gupta concluded that the majority of patients with BMS were in the age group of 51-55 years, followed by 56-60 years [19]. In a nationwide register-based study from Taiwan, Su et al. reported that female sex and increasing age were significantly associated with an increased risk of BMS during the period from 2004 to 2013 [20]. 

Hypertension was the most frequently reported condition (30.2%), followed by diabetes mellitus (24.6%). Cardiovascular disease was reported in 7.1%, kidney disease in 3.2%, and multiple systemic conditions in 4.4%, and 30.6% reported no systemic condition. The study by Gupta and Gupta found that 43.6% of BMS patients suffered from hypertension [19]. Previous studies have also reported associations between BMS and systemic illness, medication use, and subjective oral dryness. Bergdahl and Bergdahl identified age, medication, illness, subjective oral dryness, and taste disturbances among factors associated with BMS [21]. The presence of hypertension or diabetes in the present participants should not be interpreted as evidence of a causal relationship with BMS.

BMS symptoms were most commonly intermittent and often and were predominantly reported during the daytime. Ghalwash et al. reported comparable findings regarding the clinical pattern of BMS [22], whereas Gupta and Gupta observed that the burning sensation was constant throughout the day [19]. Daytime and evening symptoms were more frequently reported than morning and nighttime symptoms in the present study, broadly consistent with findings reported by Kohorst et al. [7] and Lopez-Jornet et al. [23]. The tongue was the most commonly affected site, followed by the palate, which was also consistent with previous reports [19,22].

Oral dryness was reported in 48% of the participants in the present study. A study conducted by Ghalwash et al. in the Egyptian population showed a higher rate of oral dryness (78.7%) [22]. Previous studies have reported that advanced age contributes to oral dryness among BMS patients [20]. Taste disturbance was reported in 63% of women in our study, which was higher than in previous studies [20,24]. A study conducted by Kohorst et al. reported a reduced prevalence of taste disturbances (15.9%) [7]. In the present study, 19.0% of women with BMS were categorized as having high perceived stress based on PSS-10 scores. A previous study also revealed that women attaining menopause had the highest levels of stress when compared to the other age groups [25]. A study conducted by Sarveswaran et al. showed 26% of subjects experienced stress [26]. Porporatti et al. conducted a meta-analysis specifically examining stress and found greater stress-related findings among BMS patients than controls in questionnaire-based studies, as well as differences in several stress-related biomarkers [27]. The present study does not provide a control group or an analysis directly associating PSS-10 scores with BMS occurrence; thus, a causal relationship between stress and BMS cannot be established.

Clinical implications and importance of screening

The findings of the present study have an important clinical implication for the routine oral healthcare of postmenopausal women. The relatively high frequency of BMS (26.5%) identified in this clinic-based sample in postmenopausal women suggests that oral burning symptoms should not be overlooked during routine dental and medical consultations in this population. Adamo et al. highlighted the diagnostic challenges associated with BMS, attributing delayed diagnosis partly to limited knowledge among specialists and the high frequency of misdiagnosis [28]. Postmenopausal women presenting with complaints of burning, stinging, altered oral sensation, oral dryness, or taste disturbances should undergo a focused oral assessment to identify clinically evident mucosal abnormalities and other potentially treatable local causes. A simple screening approach incorporating targeted questions regarding the presence, duration, frequency, timing, and anatomical site of oral burning, together with assessment of subjective oral dryness and taste disturbances, may facilitate earlier recognition of patients who require further evaluation. The association of BMS symptoms with subjective oral dryness and taste disturbances observed in the present study also highlights the importance of a multidisciplinary approach. Dental practitioners, oral medicine specialists, primary-care physicians, gynecologists, and other relevant healthcare professionals may have complementary roles in the assessment and management of affected women.

Limitations

This study has several limitations. First, its single-center, cross-sectional design and recruitment from an oral medicine clinical setting may have introduced referral and selection bias, limiting the generalizability of the observed frequency of BMS to all postmenopausal women. Second, systematic evaluation of all potential secondary causes of oral burning may not have been comprehensive, and diagnostic misclassification cannot therefore be completely excluded. Third, reliance on self-reported symptoms may have introduced recall and reporting bias despite the use of structured, face-to-face interviews. Finally, perceived stress was assessed only among women with BMS, without a comparison group of women without BMS. Therefore, the study describes the distribution of perceived stress among women with BMS but cannot establish an association, predictive relationship, or causal relationship between stress and BMS. The cross-sectional design also limits assessment of temporal relationships. Future multicenter, population-based, longitudinal studies with appropriate comparison groups and systematic evaluation of secondary causes are warranted to improve generalizability and further investigate factors associated with BMS.

Strengths of the study

A key strength of the study was the consecutive recruitment of all eligible postmenopausal women attending the clinic during a defined one-year period, and the use of a standardized clinical proforma, examination by a single experienced oral medicine specialist, predefined diagnostic criteria based on ICOP, and structured assessment of symptom characteristics. The study also provides clinic-based data from an Indian setting, where evidence regarding BMS among postmenopausal women remains limited.

Future recommendations

Future multicenter studies with larger and more diverse sample sizes are recommended to enhance the generalizability of the findings. Longitudinal studies evaluating the role of hormonal alterations, HRT, and their association with BMS may provide deeper insights into its pathogenesis and aid in the development of targeted therapeutic strategies.

## Conclusions

The present single-centre, clinic-based, cross-sectional study identified BMS among one-fourth of eligible postmenopausal women attending the study centre, with the highest occurrence in the 55-59 year age group, and the tongue being the most commonly affected site. Oral dryness, taste disturbances, and moderate perceived stress were commonly observed among women with BMS. Dentists, particularly oral medicine specialists, have an important role in recognizing symptoms, providing patient counselling and reassurance, and facilitating appropriate multidisciplinary care.

The cross-sectional, single-centre design, absence of a non-BMS comparison group, potential selection and self-reporting biases, and unadjusted statistical analyses limit the ability to establish causal relationships or identify independent risk factors. The findings should therefore be interpreted within the context of the study participants and potential confounding factors. Given the multifactorial nature of BMS, careful clinical evaluation and exclusion of potential local and systemic causes are important for appropriate diagnosis and management.
